# Study on anisotropy orientation due to well-ordered fibrous biological microstructures

**DOI:** 10.1117/1.JBO.29.5.052919

**Published:** 2024-02-28

**Authors:** Zhidi Liu, Jiawei Song, Qiqi Fu, Nan Zeng, Hui Ma

**Affiliations:** a Tsinghua University, Shenzhen International Graduate School, Shenzhen, China; b Tsinghua University, Shenzhen International Graduate School, Guangdong Research Center of Polarization Imaging and Measurement Engineering Technology, Shenzhen Key Laboratory for Minimal Invasive Medical Technologies, Shenzhen, China; c Tsinghua University, Department of Physics, Beijing, China

**Keywords:** polarization, anisotropic orientation, birefringence, Mueller matrix

## Abstract

**Significance:**

Most biological fibrous tissues have anisotropic optical characteristics, which originate from scattering by their fibrous microstructures and birefringence of biological macromolecules. The orientation-related anisotropic interpretation is of great value in biological tissue characterization and pathological diagnosis.

**Aim:**

We focus on intrinsic birefringence and form birefringence in biological tissue samples. By observing and comparing the forward Mueller matrix of typical samples, we can understand the interpretation ability of orientation-related polarization parameters and further distinguish the sources and trends of anisotropy in tissues.

**Approach:**

For glass fiber, silk fiber, skeletal muscle, and tendon, we construct a forward measuring device to obtain the Mueller matrix image and calculate the anisotropic parameters related to orientation. The statistical analysis method based on polar coordinates can effectively analyze the difference in anisotropic parameters.

**Results:**

For those birefringent fibers, the statistical distribution of fast-axis values derived from Mueller matrix polar decomposition was found to exhibit bimodal characteristics, which is a key point in distinguishing the single-layer birefringent fiber sample from a layered, multioriented fibrous sample. The application conditions and interference factors of anisotropic orientation parameters are analyzed. Based on the parameters extracted from the orientation bimodal distribution, we can evaluate the relative change trend of intrinsic birefringence and form birefringence in anisotropic samples.

**Conclusions:**

The cross-vertical bimodal distribution of the fast axis of anisotropic fibers is beneficial to accurately analyze the anisotropic changes in biological tissues. The results imply the potential of anisotropic orientation analysis for applications in pathological diagnosis.

## 1 Introduction

Optical anisotropy exists in most biological fibrous tissues, such as myofibrils,^1^ elastin,^2^ collagen fibers,^3^ and nerve bundles.^4^ All these tissues contain uniaxial or biaxial birefringent structures. The changes in the microstructure and orientation of these anisotropic fibers are often closely related to specific biological processes and histopathological changes.^1^^,^^5^ Since the polarization of light is highly sensitive to anisotropic microstructural organization in tissues and cells, polarization-based detection has great potential in biomedical imaging and diagnosis.^5^

Mueller matrix provides a comprehensive description of the polarization properties of the sample.^5^ By performing some mathematical transformations on the Mueller matrix, such as Mueller matrix transformation (MMT),^6^ and MMPD,^7^ some anisotropy-related physical parameters can be derived. Diattenuation is related to amplitude anisotropy (corresponds to differential attenuation of orthogonal polarization states), which can be characterized by the axis of maximum transmittance. Retardance is related to phase anisotropy (represents the phase shift between two orthogonal polarization states). Specifically, retardance includes linear retardance (LR) and circular retardance (CR). CR is derived from chiral arrangement of lamellar structures or solutions of chiral molecules, such as glucose.^8^ LR and the corresponding fast-axis orientation are related to linear birefringence in fibrous biological microstructures.^7^

LR is widely used as a parameter for evaluating the tissue microstructures (e.g., tendon,^9^ skeletal,^10^ or skin^11^) and is also helpful for the study of the mechanical properties of connective tissue,^12^ and some related pathological diagnoses (such as cirrhosis^13^ and cancer^14^). However, the linear birefringence that leads to LR can be divided into two types: one is intrinsic birefringence and the other is form birefringence.^15^ If we can identify and distinguish these two anisotropy sources, not only the mechanism of polarization characterizing tissue properties will be clear, but also the accuracy of pathological diagnosis based on polarization images will be promoted. Since both types of linear birefringence generally coexist in biological tissues, the detected LR is the coupling of intrinsic birefringence and form birefringence.^3^ Therefore, it is difficult to analyze the specific physical origin of phase anisotropy by LR.

Intrinsic birefringence originates from the order of protein molecular conformation and different chemical groups in biological tissue structures, which is similar to the properties of birefringent crystalline materials.^1^^,^^15^ Myogenic fibers and collagen fibers are cylinders with positive birefringence effect, and their fast-axis orientation is perpendicular to the long axis of the fiber.^15^ In a single muscle fiber, the contribution of the myosin subfragment is the molecular origin of intrinsic birefringence in skeletal muscle.^16^ Both sarcomere length and the disorder in the orientation of the thick and thin filament array affect the measurement results of muscle birefringence.^17^ Most collagen fibers are positively birefringent materials due to the quasicrystalline arrangement of the amino acid residues that comprise the polypeptide chains of the collagen molecule alpha chains, and therefore, have an optical fast axis that is orthogonal to the long axis of the fiber.^18^ The intrinsic birefringence in collagen tissues has also become an important factor in the study of the propagation of polarized light in biological turbid media.^19^ However, for bulk tissues, the scattering effect of the complex microstructure tends to make chaotic the fast-axis orientation corresponding to the intrinsic birefringence.^20^^,^^21^ Form birefringence arises from the anisotropic scattering effect of aligned cylinders or aligned ellipsoids immersed in media with different refractive indices. After multiple forward scattering events in fibrous tissue, an increase in LR is introduced between orthogonal polarization basis vectors.^18^ Early researchers designed a scattering model combined with Monte Carlo simulations and found that the fast axis of cylindrical scatterers is parallel to the long axis of the cylinder.^22^^,^^23^ By backscattering polarimetric imaging of skeletal muscles and tendons, He et al.^24^ demonstrated that scattering in biological fibers contributes significantly to anisotropy and further studied anisotropy orientations originated from back scattering and birefringence of turbid media using Mueller matrix-derived parameters.^25^ Since most of the fibers in biological tissues are cylindrical structures with positive birefringence, there should exist a pair of mutually orthogonal fast axes corresponding to intrinsic birefringence and form birefringence, respectively. Thus the statistical distribution characteristics of the fast-axis orientations of tissue samples may provide a way of identifying and distinguishing birefringence sources.

In this work, we built a forward Mueller matrix imaging system to measure the Mueller matrix of biological samples with fibrous structures including silk, skeletal muscle, and tendon. Then we calculate the parameters related to anisotropic orientation by MMPD and MMT and compare their statistical distributions. In the statistical distribution of the fast-axis orientation of the well-ordered fiber tissue, we observed two major peaks that are perpendicular to each other. Both the external tension on the fiber tissue and its own internal scattering can influence the relative strength of two peaks. We extracted new parameters from the statistical distribution of fast axis to assist the evaluation of intrinsic and form birefringence. The results also imply the potential of anisotropic orientation analysis for applications in biological tissue characterization.

## 2 Methods and Materials

### 2.1 Experimental Setup

**Fig. 1:**
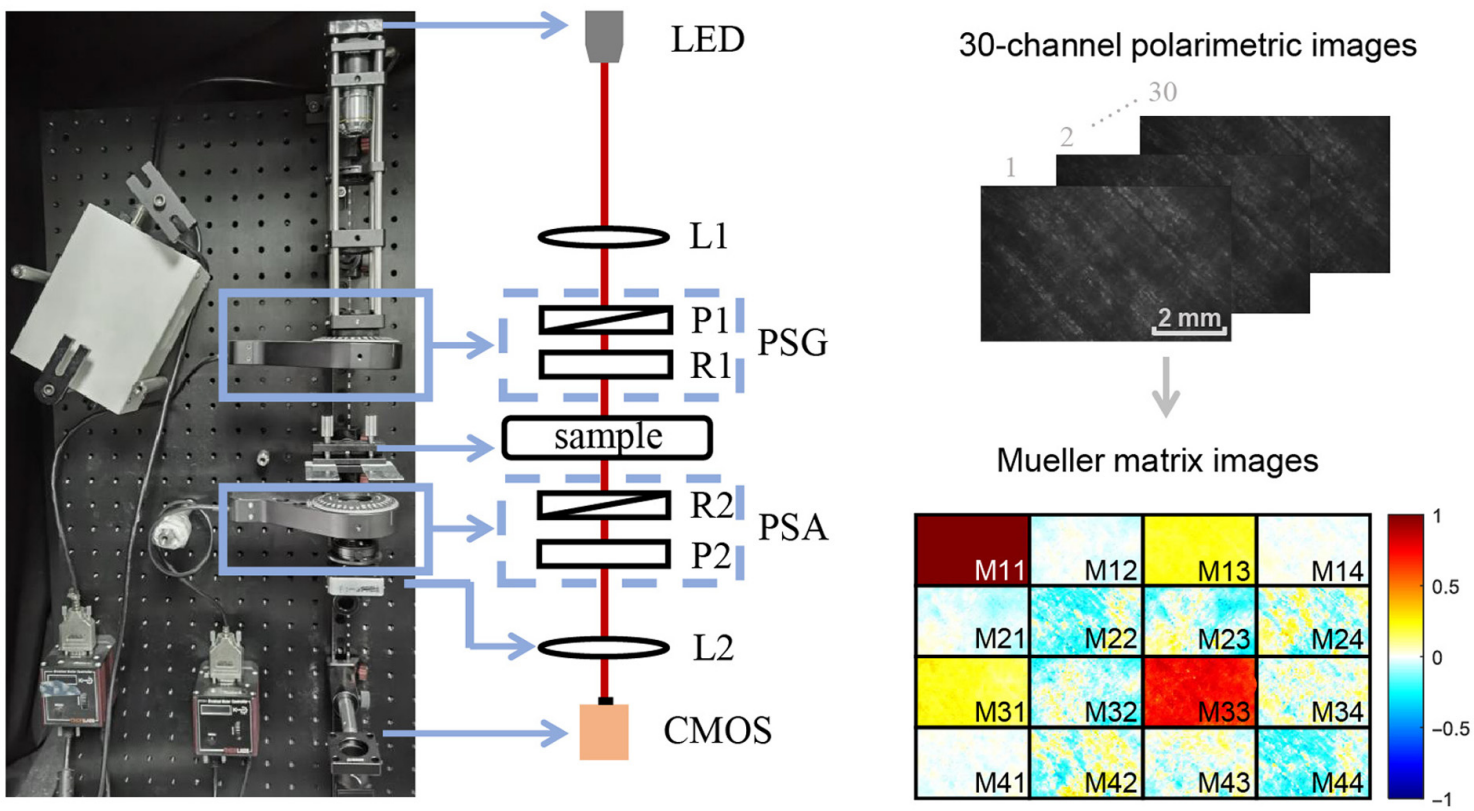
Photo and schematic of the Mueller matrix imaging system. P1 and P2: polarizer; R1 and R2: quarter-wave plate; L1: collimating lens; L2: imaging lens.

Figure 1 shows the Mueller matrix imaging system on basis of the dual-rotating quarter-wave plate (QWP) method.^25^ The illumination light from LED (3 W, 633 nm, Δλ=20  nm, Cree, China) passes through the polarization state generator (PSG) and then generates an incident polarization state, which is illuminated on the sample. Then the signal light after passing through the sample is received by a CMOS (MVCA023-10UM, Hikvision, China) through the polarization state analyzer (PSA) and an imaging lens (L2, f=80  mm, NA = 0.16, Daheng Optics, China). Both PSG and PSA are composed of a fixed polarizer (P1 and P2, LPVISE100-1, Thorlabs, Inc.) and a QWP (R1 and R2, Daheng Optics, China). The fast-axis direction of all wave plates and the transmission axis of the polarizer are parallel to each other in the initial state. During the experiment, the computer controlled R1 and R2 to rotate 30 times synchronously at the step speed of 6 deg and 30 deg, respectively, and the corresponding 30-channel polarimetric image was detected by CMOS camera. Then a Mueller matrix image can be calculated from these 30-channel polarimetric images based on a Fourier relationship.^26^ Image registration technique and an air calibration algorithm^27^ are also used to reduce measurement errors in the process. After collecting the Mueller matrices of the air, the system is calibrated by the numerical calibration method, and the maximum error is <0.01.

### 2.2 Polarization Parameters Derived from Mueller Matrix

Mueller matrix imaging methods have shown promising results in detecting wavelength-scale microstructures and improving the diagnosis of pathological samples.^21^ Previous researchers decoupled many physically meaningful and independent parameters from the Mueller matrix via MMPD and MMT.^6^^,^^7^ In this study, we selected θ, $\alpha_{P}$, and $\alpha_{q}$ from these parameters to analyze the anisotropic orientation of biological samples, as shown in Eqs. (1)–(3). The parameter θ from MMPD, shown as Eq. (1), represents the orientation angle of the fast axis corresponding to LR of the sample, where $m_{R}$ is the 3×3 submatrix of an LR matrix, and δ is the magnitude of LR.^7^ θ is closely related to LR, where LR is the phase difference between two eigenpolarization vectors, and θ is the electric field vibration direction of the phase advanced eigenpolarization vector. The $\alpha_{P}$ and $\alpha_{q}$ from MMT, as shown in Eqs. (2) and (3), represent the orientation angles related to diattenuation and birefringence, respectively. The parameter $\alpha_{P}$ is the azimuth angle of the linear diattenuation effect, and its physical meaning is that the transmittance is maximum when the light passing through the sample with the vibration direction of the electric field vector parallel to $\alpha_{P}$. $\alpha_{q}$ is the azimuth angle calculated according to $m_{42}$ and $m_{43}$, which are strongly related to birefringence and represent the ability to convert linearly polarized light into circularly polarized light. When sample has a pure LR effect, $\alpha_{q}$ represents the direction of the optical fast axis:

$$\theta =\frac{1}{2}\;\arctan(r_{2}/r_{1}),\;r_{i}=\frac{1}{2\;\sin\;\delta }\times \overset{3}{\underset{j,k}{\sum }}\varepsilon_{ijk}m_{R}(j,k),$$ (1)

$$\alpha_{P}=\frac{1}{2}\;\arctan(m_{31}/m_{21}),$$ (2)

$$\alpha_{q}=\frac{1}{2}\;\arctan(m_{42}/-m_{43}).$$ (3)

Moreover, the depolarization parameter Δ derived from MMPD is used to quantify the effect of scattering, and the formula is shown as Eq. (4), where $m_{\Delta }$ is the 3×3 depolarization submatrix resulting from MMPD, tr() represents the trace of the matrix:

$$\Delta =1-|\mathrm{tr}(m_{\Delta }-1)|/3.$$ (4)

**Fig. 2:**
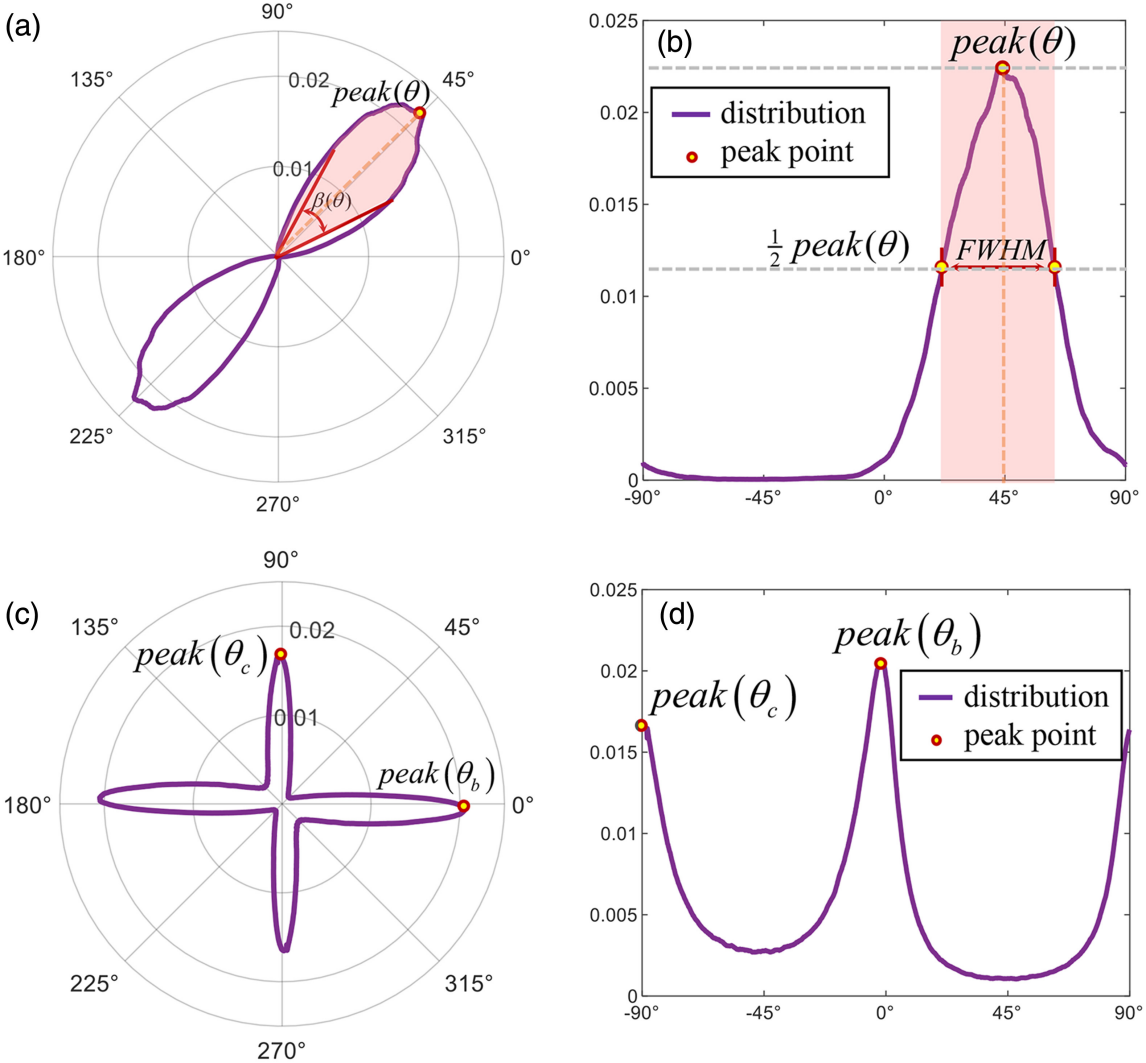
FDH schematic: (a), (b) the single-peaked FDH and (c), (d) the double-peaked FDH in polar and Cartesian coordinate systems, respectively.

As shown in Fig. 2, the frequency distribution histogram (FDH) has always been a useful tool for analyzing statistical polarization information. In our previous work, the differences in statistical distributions were observed to provide auxiliary information for judging changes in the microstructure.^14^^,^^21^ Recently, we found that the fibrous sample containing birefringence will show two peaks on the FDH of polarization parameters θ. For visual intuition, here we plot the FDHs of polarization parameters θ, $\alpha_{P}$, and $\alpha_{q}$ in polar coordinates in this work. For a single-peaked distribution, Fig. 2(a) shows the orientation distribution in polar coordinates, which is symmetric with 180 deg as the periodic center, which is equivalent to the FDH in the Cartesian coordinate system shown in Fig. 2(b). Moreover, the visualization effect of the double peak in the Cartesian coordinate system in Fig. 2(d) is obviously not as intuitive as Fig. 2(c). In order to further extract information from FDH, we define a function peak() to represent the probability density occupied by the peak angle, and the parameter β(θ) to represent the range of angle fluctuation around the peak angle, which meaning is equivalent to the FWHM in the Cartesian coordinate system. The formula of β(θ) is shown as

$$\beta (\theta )={\mathrm{fdh}}^{-1}{\left[\frac{1}{2}\text{peak}(\theta )\right]}_{\max}-{\mathrm{fdh}}^{-1}{\left[\frac{1}{2}\text{peak}(\theta )\right]}_{\min}.$$ (5)

Usually, we only use the formula to calculate an average orientation angle θ of the sample from its polarization images, when this two-peak histogram distribution may be missed. In this work, the tissue-related characterization information will be extracted from the peak distribution in a polar coordinate system. By referring to our previous research,^28^ both birefringence and fibrous scatterers will cause tissue anisotropy and generate the phase retardance. For the FDH of θ, we assume that the peak angle corresponding to form birefringence is $\theta_{c}$, and the peak angle corresponding to intrinsic birefringence is $\theta_{b}$. We define γ to represent the share of $\theta_{b}$ in the total statistical weight of the two peak angles, and the formula is shown as

$$\gamma =\text{peak}(\theta_{b})/[\text{peak}(\theta_{c})+\text{peak}(\theta_{b})].$$ (6)

### 2.3 Samples

**Fig. 3:**
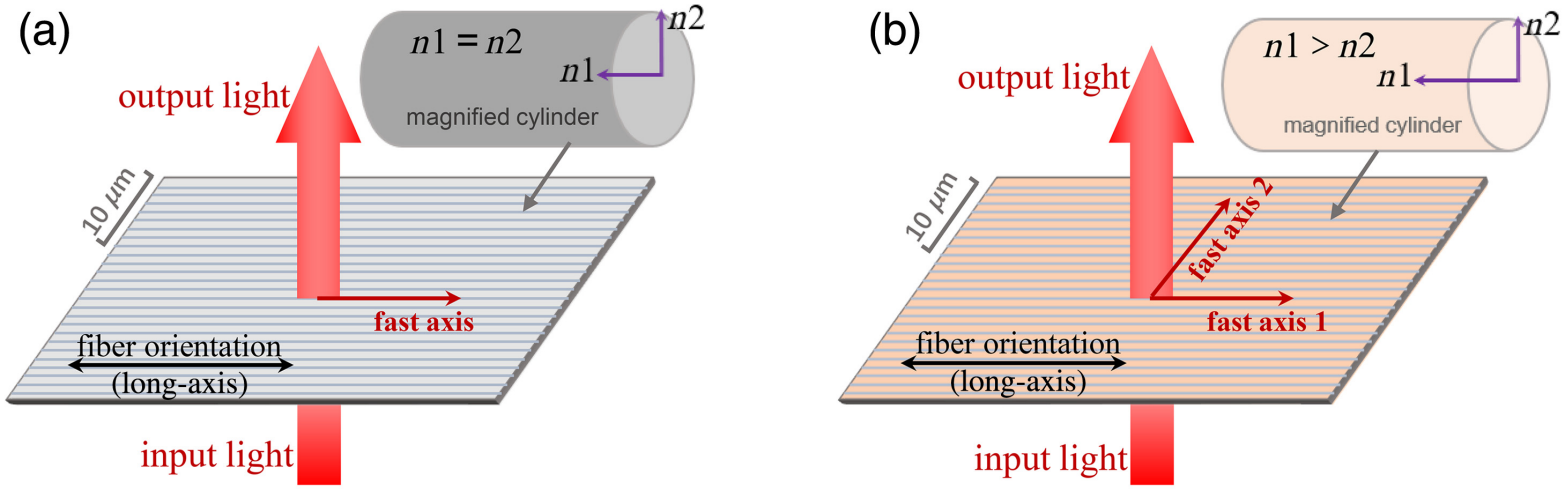
Schematic diagram of the fast-axis orientation of cylindrical fibers. (a) Fast-axis orientation of nonbirefringent cylinders with axial refractive index $n_{1}$ equal to radial refractive index $n_{2}$. (b) Double fast-axis orientation of birefringent cylinders with axial refractive index $n_{1}$ greater than radial refractive index $n_{2}$.

For neatly aligned fibrous microstructures, we classify them into two types depending on whether the fibers have an intrinsic birefringence effect or not. The first case, as shown in Fig. 3(a), consists of cylinders without intrinsic birefringence effect, and the axial and radial refractive indices of the cylinders are equal. The second case, shown in Fig. 3(b), consists of cylinders with positive birefringent properties, and the axial refractive index of the cylinders is greater than the radial refractive index. Such cylindrical microstructures are more common in biological tissues, where the intrinsic birefringence can be due to the ordered lattice of protein molecules in the fibers.

**Fig. 4:**
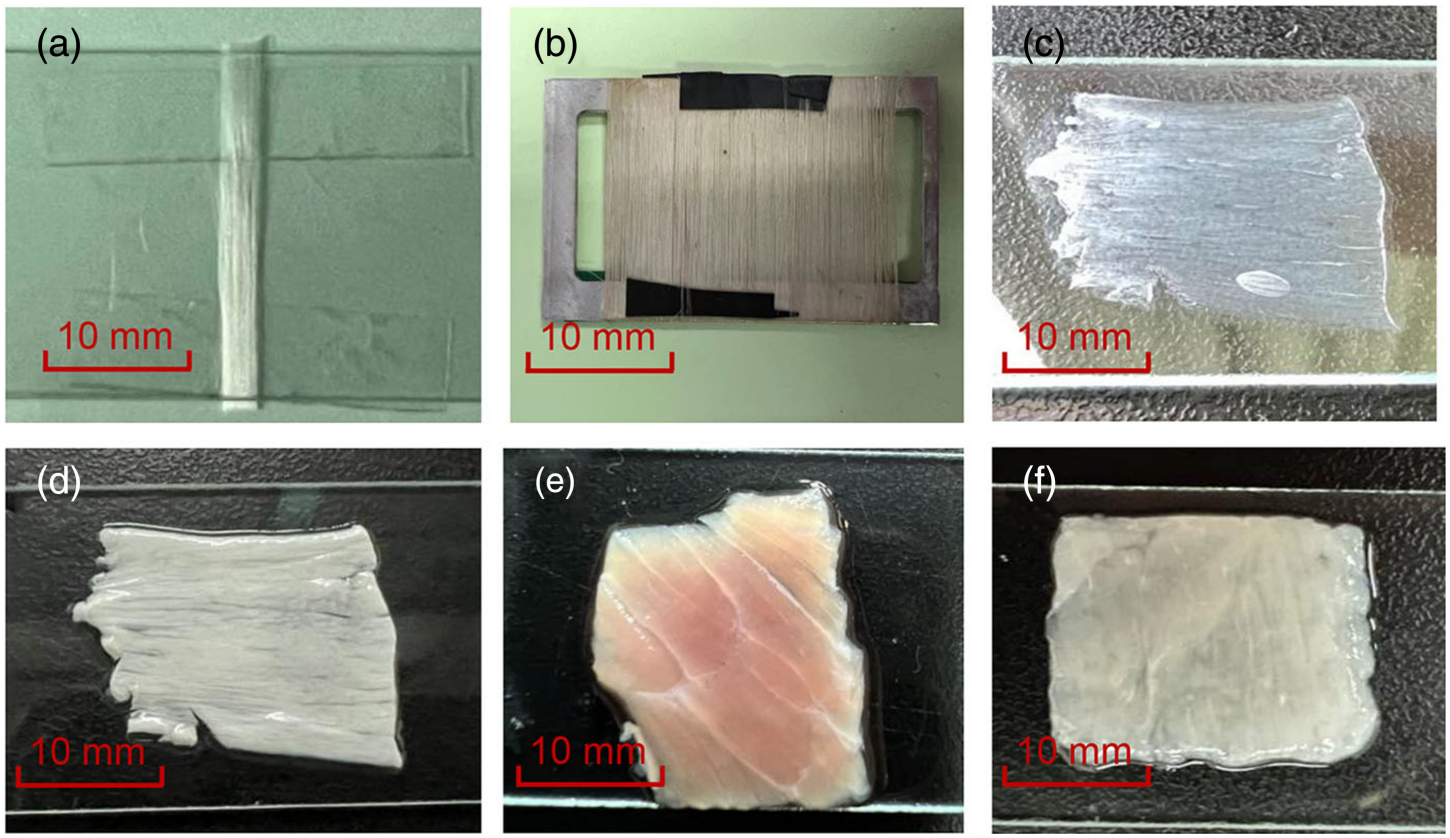
Samples used in the experiments: (a) neatly oriented glass fibers, (b) neatly oriented silk, (c) air-dried bovine tendon, (d) fresh bovine tendon, (e) fresh bovine skeletal muscle, and (f) fresh chicken breast.

Photographs of the samples used in the experiments are given in Fig. 4. In this work, we chose glass fibers (2400TEX-180, Hongtu Composite Materials Co., Ltd, China) and silk (provided by Guangxi Institute of Supervision and Testing on Product Quality) as typical scatterers, to verify the anisotropic orientation properties of nonbirefringent and birefringent cylinders, as shown in Figs. 4(a) and 4(b). The glass fiber is made from nonbirefringent materials with a 1.547 refractive index, and its radius is 5  μm observed by scanning electron microscope (SEM).^25^ Silk is a birefringent cylindrical scatterer with axial refractive index $n_{1}=1.57$ and radial refractive index $n_{2}=1.52$, which can be equated to a positive uniaxial crystal with its fast-axis oriented perpendicular to the long axis. The diameter of a single strand of silk is 30  μm and its internal structure consists of filamentous substructures with a diameter of about 1.5  μm (measured by SEM). In the preparation of phantom samples, the stiffer glass fibers are arranged one by one intentionally in an orderly fashion and fixed with glue. For silk fibers, they are wound neatly side-by-side on alloy supports. By winding different layers of silk on the alloy scaffold, we can make phantoms with birefringent cylindrical scatters and change their scattering intensity.

Considering that collagen fibers and myogenic fibers are two birefringent fibrous tissues commonly found in animals, tendons and skeletal muscles were used in our experiments. The tendon was obtained from the hoof part of the cow, and the main component was collagen fibers. Figure 4(c) shows the air-dried tendon, which resembles a near-transparent purely birefringent sample due to refractive index matching after air drying. Figure 4(d) shows the fresh tendon, which has a strong anisotropic scattering effect and birefringence effect. Its opaque, turbid white appearance is due to Mie scattering of fibrous microstructures. Figure 4(e) shows a fresh bovine skeletal muscle sample, which is used to compare with tendon in terms of anisotropic orientation. Figure 4(f) shows a chicken breast sample, used in muscle stretching experiments, which contains less fat and fascia and has more neatly arranged fibers.

## 3 Results

### 3.1 Anisotropic Orientation Characteristics of Neatly Aligned Fiber Samples

The polarization effects of birefringent cylinder arising from the coupling of anisotropic scattering and lattice birefringence are relatively complex. Therefore, we first studied samples of nonbirefringent cylinders with no coupling effect (i.e., glass fiber) and samples of pure birefringent (i.e., air-dried tendons) to clarify the relationship between the three orientation parameters and the actual fiber orientation. Then we studied the orientation characteristics of the birefringent cylinder samples (i.e., silk). We found that the physical meaning of the two peaks in the FDH of θ can be distinguished according to $\alpha_{P}$.

#### 3.1.1 Anisotropic orientation of nonbirefringent cylindrical samples and pure birefringent samples

**Fig. 5:**
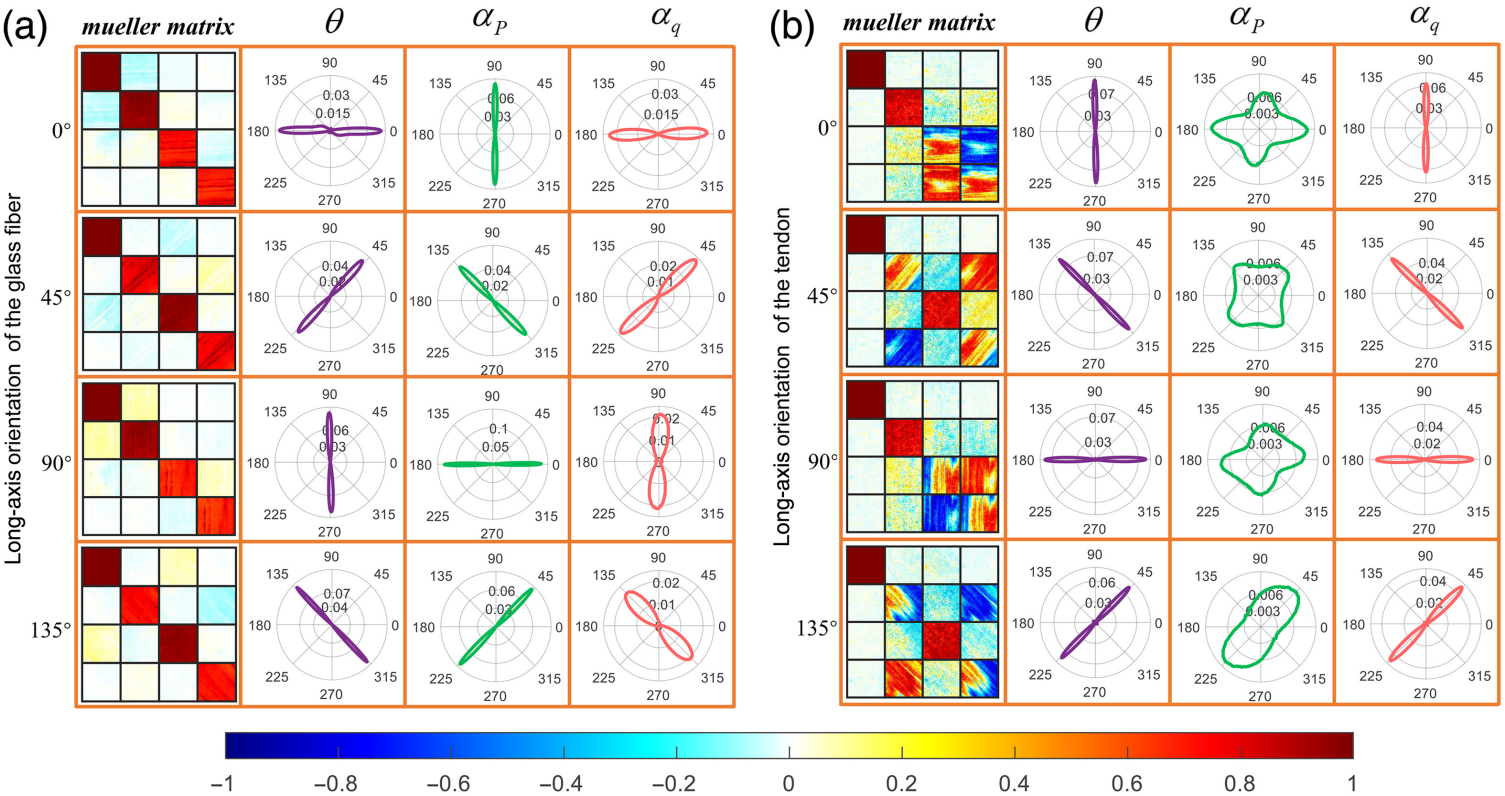
Mueller matrix images and anisotropic orientation parameters of samples at different spatial orientations: (a) well-aligned glass fibers, (b) air-dried tendon (thickness: 300  μm). Each subimage is divided into 4×4 areas by an orange grid, and the top to bottom rows correspond to the four spatial orientations of the sample at 0 deg, 45 deg, 90 deg, and 135 deg; the left to right columns correspond to the Mueller matrix images of the measured sample and the FDH of the three parameters θ, $\alpha_{P}$, and $\alpha_{q}$ in polar coordinates. All Mueller matrix images share the same color bar.

Figure 5 shows the anisotropic orientation of pure anisotropic scattering and pure birefringence by measuring glass fibers and air-dried tendons, respectively. In Fig. 5(a), all three parameters can track the orientation of the fibers, indicating that nonbirefringent cylindrical scatters produces both diattenuation and form birefringence. $\alpha_{P}$ is perpendicular to the fiber long axis, meaning that the electric field vector component with the vibration direction perpendicular to the glass fiber axis has a higher transmittance. θ is consistent with the glass fiber axis, which means that the fast axis corresponding to the form birefringence induced by anisotropic scattering is parallel to the fiber long axis. $\alpha_{q}$ is parallel to the fiber long axis, also indicating that the scattering by cylinders causes an LR. In Fig. 5(b), the tendon becomes nearly transparent after air-drying, and its anisotropy is dominated by the intrinsic birefringence of the collagen fibers, while the anisotropic scattering effect is reduced. Therefore, $\alpha_{P}$ cannot track the orientation of collagen fibers. The fast-axis orientation by θ is perpendicular to the collagen fibers’ long axis, which also confirms that collagen fibers have a positive birefringence effect and is in agreement with the conclusions of previous researchers.^18^^,^^19^ $\alpha_{q}$ is perpendicular to the fiber long axis, meaning that the intrinsic birefringence causes an LR. For details of quantitative values of diattenuation and LR, refer to Tables S1 and S2 in the Supplementary Material.

#### 3.1.2 Anisotropic orientation of birefringent cylindrical samples

**Fig. 6:**
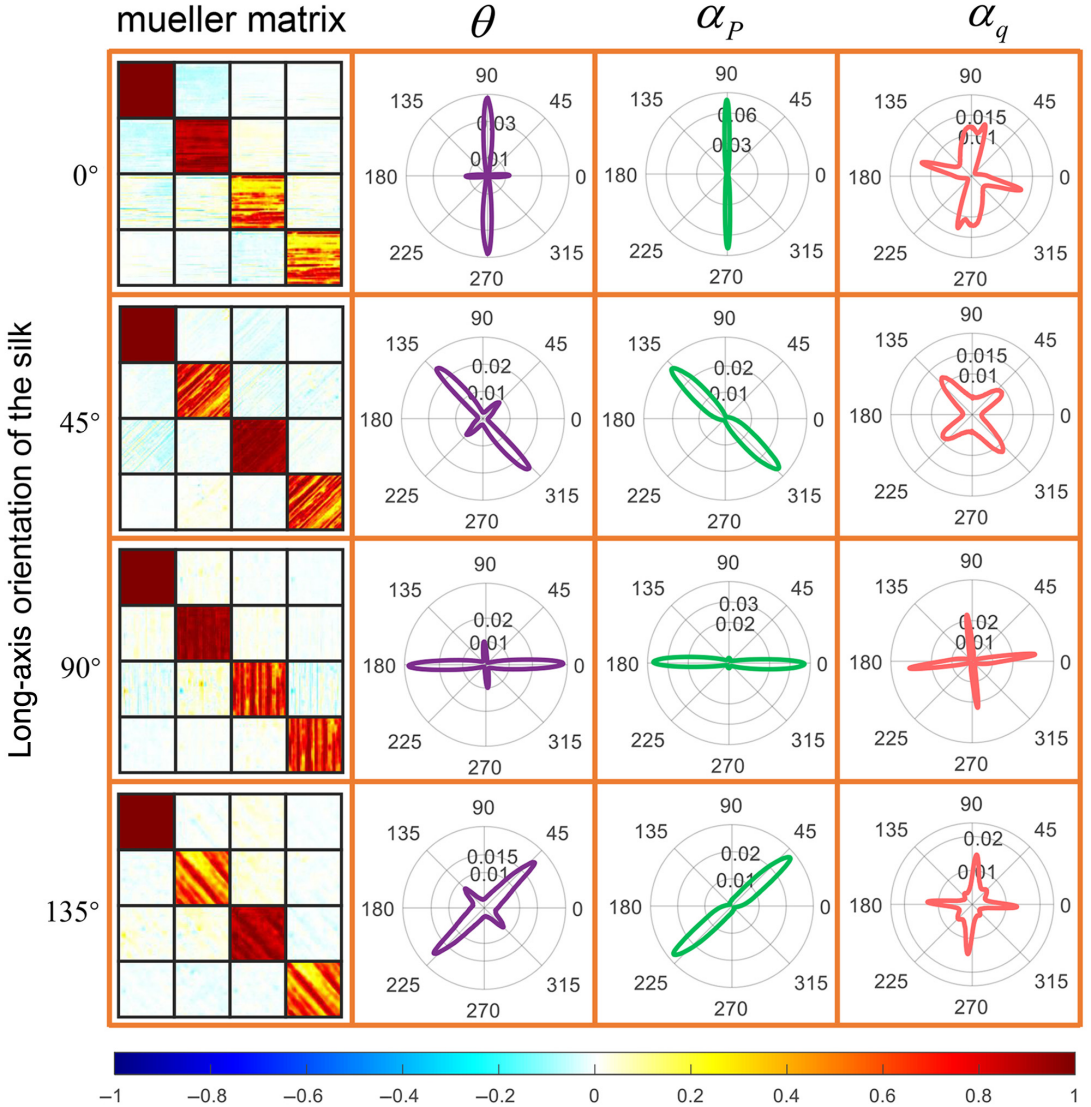
Mueller matrix images and anisotropic orientation parameters of silk at different spatial orientations. This figure is divided into 4×4 areas by an orange grid, and the top to bottom rows correspond to the four spatial orientations of the sample at 0 deg, 45 deg, 90 deg, and 135 deg; the left to right columns correspond to the Mueller matrix images of the measured silk and the FDH of the three parameters θ, $\alpha_{P}$, and $\alpha_{q}$ in polar coordinates. All Mueller matrix images share the same color bar.

The phantom is prepared by neatly winding two layers of silk along the same orientation on the alloy scaffold. We measured their Mueller matrix images and calculated the FDH of three anisotropy parameters θ, $\alpha_{P}$, and $\alpha_{q}$, as shown in Fig. 6. First, the FDH of θ shows a bimodal distribution with two peak angular orientations perpendicular to each other, which confirms that silk is a fiber with intrinsic birefringence. The fiber axial peak is due to cylindrical scattering, and the peak perpendicular to the fiber axis is due to intrinsic birefringence of the silk. By comparing the probability densities of the two peaks, we can see that the intrinsic birefringence of this two-layer silk sample contributes more to the anisotropy than the cylindrical scattering. Second, the FDH of $\alpha_{P}$ shows a unimodal distribution, implying that this parameter is only related to the diattenuation caused by cylindrical scattering. The peak angle of $\alpha_{P}$ is perpendicular to the long axis of silk. Compared with the $\alpha_{P}$ of glass fiber in Fig. 5(a), the $\alpha_{P}$ of both glass fiber and silk is perpendicular to the fiber axis. So $\alpha_{P}$ is not affected by the intrinsic birefringence and robust in characterizing fiber orientation. Third, compared with Fig. 5(a), the $\alpha_{q}$ of glass fiber and silk is not consistent for the same fiber orientation, implying that $\alpha_{q}$ is not robust in characterizing fiber orientation. For fibers without intrinsic birefringence, $\alpha_{q}$ is only related to the form birefringence caused by cylindrical scattering, and the peak angle of $\alpha_{q}$ is parallel to the fiber axis. For fibers with intrinsic birefringence, $\alpha_{q}$ will be affected by both form and intrinsic birefringence. When multiple anisotropic effects are superimposed in the sample, the parameter $\alpha_{q}$ becomes unstable and cannot effectively characterize the anisotropic orientation. Therefore, we preferentially use θ and $\alpha_{P}$ when discriminating anisotropy of biological tissues. The fast axis corresponding to form birefringence is parallel to the fiber axis, which can be determined by the orientation perpendicular to $\alpha_{P}$. Then we can distinguish the peak due to form birefringence in the bimodal FDH of θ, and the other peak corresponds to intrinsic birefringence. In addition, the fluctuation range of peak angle of FDH for θ and $\alpha_{P}$ is given in Table 1. For details of quantitative values of diattenuation and LR, refer to Table S3 in the Supplementary Material.

**Table 1:** Fluctuation range of peak angle of FDH for anisotropic orientation parameters of silk.

Long-axis orientation (deg)	Range of angle fluctuation around peak angle		
	$\beta (\theta_{b})$ (deg)	$\beta (\theta_{c})$ (deg)	$\beta (\alpha_{P})$ (deg)
0	12.33	15.03	13.23
45	18.81	21.69	25.12
90	13.32	13.50	22.59
135	20.71	26.82	18.45

$\theta_{b}$ and $\theta_{c}$ are the peak angle corresponding to intrinsic birefringence and form birefringence in the FDH of θ, respectively.

### 3.2 Study of Tissue Anisotropy Using Bimodal Distribution of Fast Axis

#### 3.2.1 Evaluating tissue mechanical properties of skeletal muscle

**Fig. 7:**
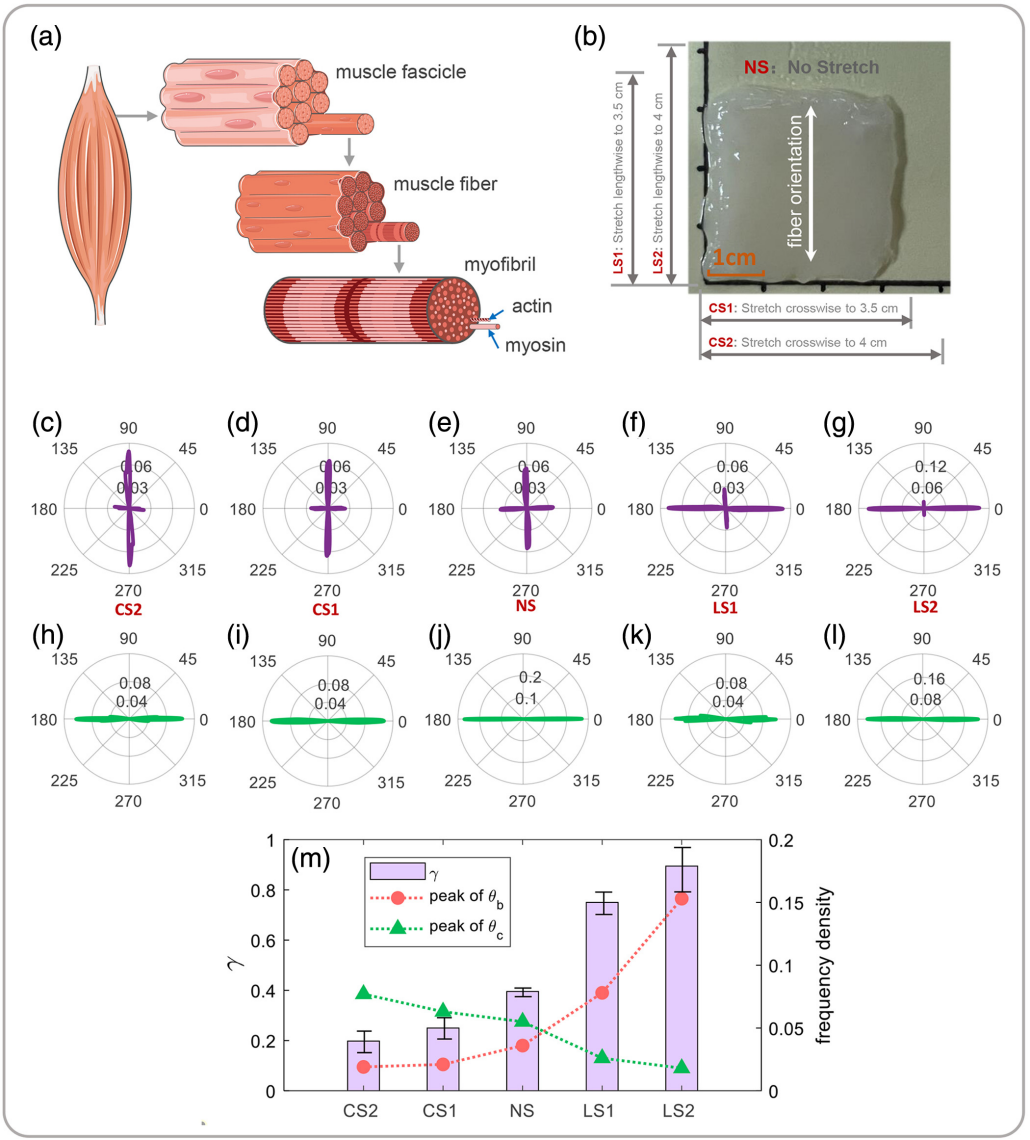
Experimental results of chicken breast under different stretching states: (a) schematic diagram of skeletal muscle anatomy. (b) Schematic diagram of muscle stretching state, NS corresponding to the initial state without stretching, CS1 and CS2 corresponding to transverse stretching to 3.5 and 4 cm, LS1 and LS2 corresponding to longitudinal stretching to 3.5 and 4 cm, respectively. (c)–(g) FDH of θ corresponding to different muscle stretching states. (h)–(l) FDH of $\alpha_{P}$ corresponding to different muscle stretching states. (m) The $\text{peak}(\theta_{b})$, $\text{peak}(\theta_{c})$ (corresponds to the right ordinate), and γ value (corresponds to the left ordinate) corresponding to different muscle stretching states.

Skeletal muscle tissue is widely distributed in mammals, and its anatomical structure is shown in Fig. 7(a). The basic unit of skeletal muscle is the myofibrils, and the fast axis corresponding to intrinsic birefringence is oriented perpendicular to its long axis. There are periodic sarcomeres in myofibrils,^15^^,^^16^ which are composed of actin and myosin. Myosin forms the thick myofilaments with birefringence, actin forms the thin myofilaments without birefringence, and the two types of myofilaments form isotropic and anisotropic bands in sarcomere. When the muscle contracts, the sarcomere becomes shorter, and when the muscle is relaxed, the sarcomere becomes longer. Here we use transverse and longitudinal stress stretching to achieve sarcomere length variation.^12^

A chicken breast slice (size: 3  cm×3  cm, thickness: 800  μm) was used for the experiment considering its lower fat content and less fascia between the muscle fibers. As shown in Fig. 7(b), the actual orientation of muscle fibers is in the vertical direction. Figures 7(h)–7(l) show the FDHs of $\alpha_{P}$ corresponding to different stretching states. The peak center angle is always 0 deg, perpendicular to the actual orientation of muscle fibers, indicating that external stretching does not affect the accuracy of $\alpha_{P}$ in fiber orientation tracking. Figures 7(c)–7(g) show the bimodal distribution characteristic in the FDHs of θ corresponding to different stretching states. Here 90 deg is the fast-axis angle corresponding to form birefringence, and 0 deg is the fast-axis angle corresponding to intrinsic birefringence. Figure 7(e) corresponds to the initial state (NS) without stretching. In the first case, from NS to CS2, the transverse stretch is strengthened, with the peak at 90 deg increasing and the peak at 0 deg decreasing. In the second case, the longitudinal stretch is strengthened, with the peak of 90 deg decreasing and the peak of 0 deg increasing. This is also confirmed by the trend of $\text{peak}(\theta_{b})$, $\text{peak}(\theta_{c})$, and γ in Fig. 7(m). These results are consistent with the myofilament sliding theory.^15^ The birefringence of muscle tissue varies with sarcomere length because some of the contractile proteins change their molecular conformation or orientation. When skeletal muscle is stretched laterally, the sarcomere becomes shorter, more actin filaments are mixed into myosin filaments, the number of overlapping fibers per unit length increases, and the form birefringence caused by anisotropic scattering is enhanced. When skeletal muscle is stretched longitudinally, actin filaments are separated from myosin and the number of overlapping fibers per unit length will decrease as the length of the sarcomere increases, reducing the anisotropy caused by cylindrical scattering. Due to sarcomere stretching, the lattice spacing of myosin molecules becomes smaller, which can increase the anisotropy caused by molecular birefringence. Obviously, this experiment also confirms the potential of the orientation analysis of polarization parameters applied to evaluate stress state of skeletal muscles. For details of quantitative values of diattenuation and LR, refer to Table S5 in the Supplementary Material.

#### 3.2.2 Evaluating anisotropy differences from different tissue types

Tendons are the connective tissue that links skeletal muscle to bone. Tendon is composed of collagen fibers, different from myofibrils in molecular structure. Collagen fibers have uniformly distributed positive birefringence effects, while myofibrils contain sarcomeres that periodically appear as “A band” and “I band.” Using the anisotropic orientation parameters to distinguish these two kinds of tissues can provide a valuable reference to further understand the optical properties of the microstructure of complex biological tissues.

##### Comparison between bovine skeletal muscle and bovine tendon with the same thickness

**Fig. 8:**
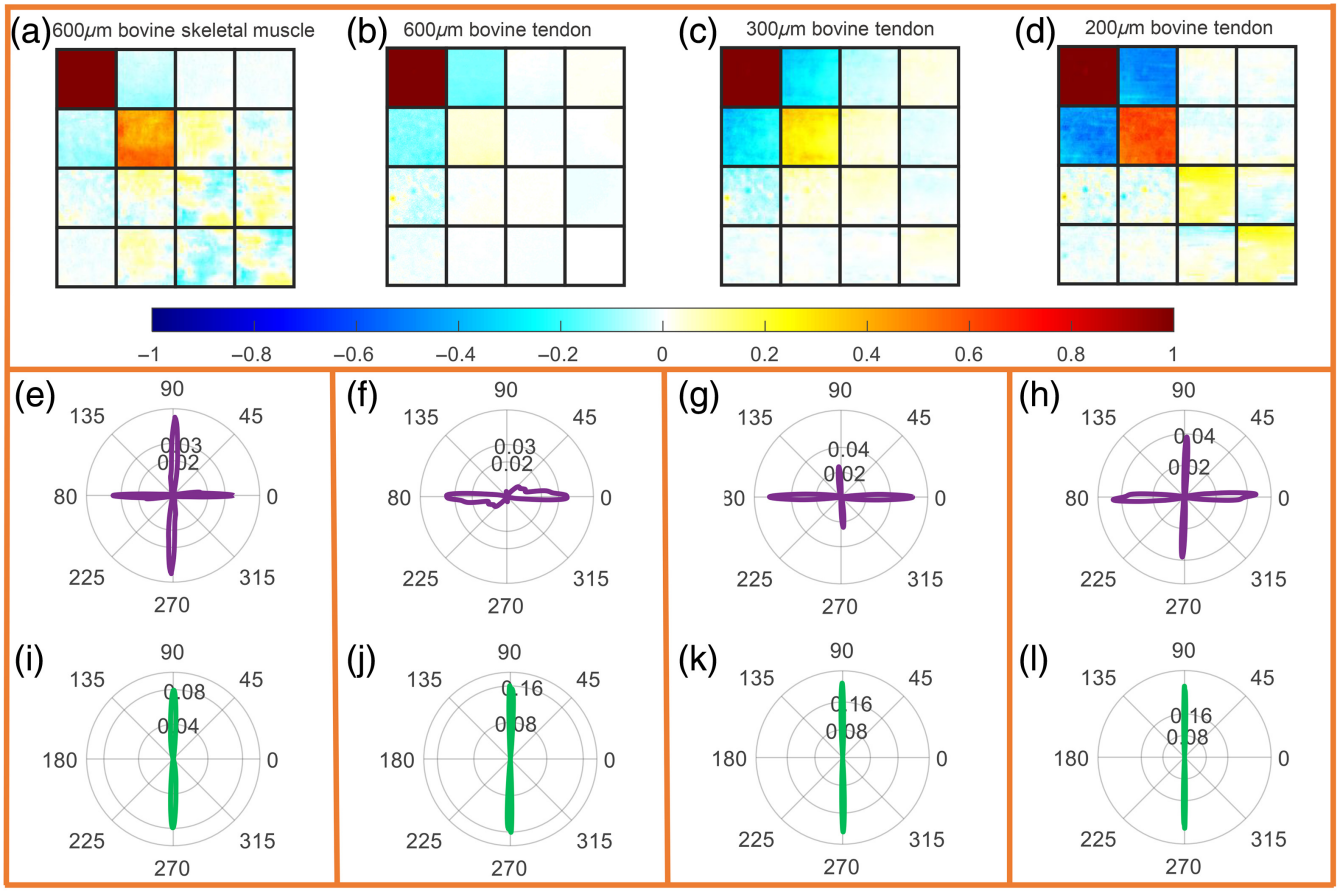
Experimental results of bovine skeletal muscle and bovine tendon. In each measurement, the actual long axis of the sample was 0 deg. The measured samples were bovine skeletal muscle with a thickness of 600  μm, and bovine tendon with a thickness of 600, 300, and 200  μm, respectively. (a)–(d) The corresponding Mueller matrix; (e)–(h) FDH of the corresponding θ; and (i)–(l) FDH of the corresponding $\alpha_{P}$. For details of quantitative values of diattenuation and LR, refer to Table S6 in the Supplementary Material.

Figures 8(a) and 8(b) show Mueller matrices of bovine skeletal muscle and bovine tendon with both 600  μm thickness. The 3×3 elements in the lower right corner of the matrix have obvious differences. The absolute values of M22, M33, and M44 of the bovine tendon are closer to 0, due to the stronger depolarization by the anisotropic scattering of the bovine tendon. In Figs. 8(i) and 8(j), both the center angle of the $\alpha_{p}$ FDH of bovine skeletal muscle and bovine tendon is 90 deg, perpendicular to the actual long axis of the fiber. The $\alpha_{p}$ FDH of skeletal muscle is wide and has a lower peak value around 0.08, whereas the $\alpha_{p}$ FDH of tendon is narrow and has a higher peak value around 0.16. These phenomena suggest that the alignment of fibers in tendons is better than in skeletal muscle. Figures 8(e) and 8(f) show the θ FDH of bovine skeletal muscle and bovine tendon, whose distribution characteristics are obviously different. Skeletal muscle’s bimodal FDH confirms that it contains both form birefringence and intrinsic birefringence. In contrast, the tendon only shows a single peak at 0 deg, corresponding to the form birefringence caused by anisotropic scattering, while the intrinsic birefringence is obliterated by multiple scattering.

##### Comparison of tendon tissue of different thickness

We further measured tendons with thinner thickness. Figures 8(c) and 8(d) show Muller matrix images of tendon slices with thickness of 300 and 200  μm, respectively. Figures 8(k) and 8(l) indicate that the central angle of $\alpha_{P}$ FDH of tendon is always 90 deg not affected by sample thickness. In Figs. 8(f)–8(h), we can see that the FDHs of θ changed from a single peak to bimodality with the decreasing tendon thickness, and the 90 deg peak corresponding to intrinsic birefringence gradually became apparent. This indicates that with the increase of fiber thickness, the intrinsic birefringence information of biological fiber itself will be covered by multiple forward scattering of the cylinders. When the slice thickness of the sample is thicker, the scattering effect is stronger, and the intrinsic birefringence is not easy to measure.

## 4 Discussion

### 4.1 Influence of Increasing Fiber Scatterers on Bimodal Distribution Characteristics

For forward imaging, light is simultaneously scattered and transmitted as it penetrates the sample. That is, the scattering of photons is affected by the form birefringence, and the transmission of photons contains information about the intrinsic birefringence. However, multiple scattering and transmission processes alternate and interact with each other. Focusing on samples containing birefringent fibers, we study the influence of cylindrical scatterers on optical fast axis. It is found that the increasing scattering by cylinders can change the fast-axis bimodal distribution.

**Fig. 9:**
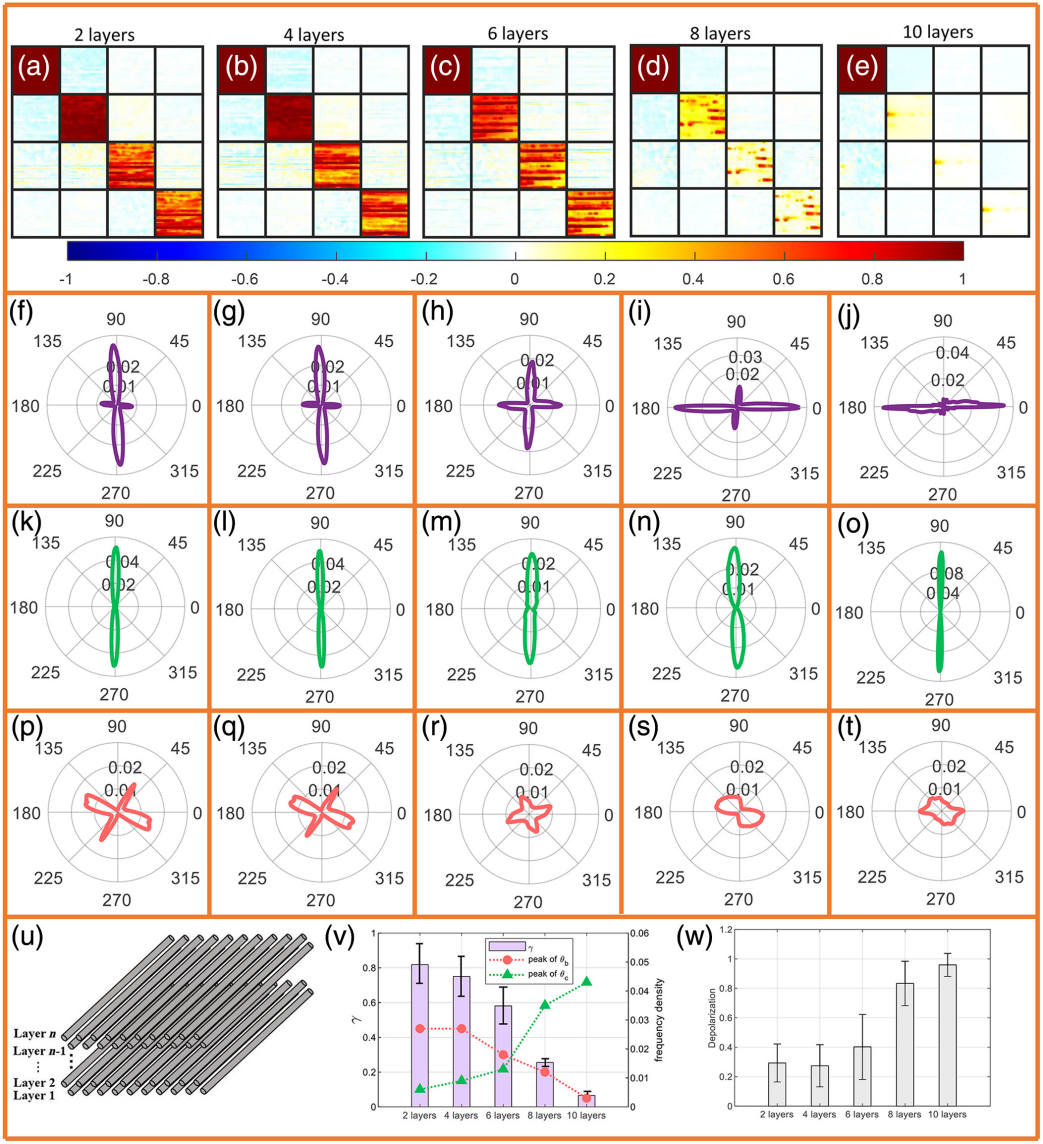
Experimental results for neatly arranged silk of different thicknesses. The thicknesses of the silk are 2, 4, 6, 8, and 10 layers, respectively. (a)–(e) The corresponding Mueller matrix images; (f)–(j) the corresponding FDH of θ; (k)–(o) the corresponding FDH of $\alpha_{P}$; (p)–(t) the corresponding FDH of $\alpha_{q}$; (u) a schematic representation of the silk sample; (v) the $\text{peak}(\theta_{b})$, $\text{peak}(\theta_{c})$ (corresponds to the right ordinate), and γ value (corresponds to the left ordinate) of silk with different layers; and (w) is the depolarization value corresponding to different layers of silk. In each measurement, the actual long axis of the silk was 0 deg.

Figures 9(a)–9(e) show the Mueller matrix images of silk samples with different layers, and Fig. 9(w) shows the corresponding depolarization values. It is reasonable that the increased silk layers will strengthen the depolarization continuously. Figures 9(k)–9(o) show that the $\alpha_{P}$ FDHs of silk samples with different layers always present a single peak with the center angle of 90 deg, confirming that $\alpha_{P}$ has a high robustness under different scattering intensities. Figures 9(f)–9(j) show the θ FDHs of the silk with different layers. The peak angle 0 deg corresponding to form birefringence is increasing, and the peak angle 90 deg corresponding to intrinsic birefringence is decreasing. This is also confirmed by the trend of $\text{peak}(\theta_{b})$, $\text{peak}(\theta_{c})$, and γ in Fig. 9(v). These experimental results show that with the increasing birefringent fiber content, the fast-axis orientation will be gradually dominated by cylindrical scattering, and the information from intrinsic birefringence of a single fiber will be lost in the process of multiple scattering. This is consistent with the experimental results of tendon slices of different thickness in Sec. 3.2.2. However, as shown in Figs. 9(p)–9(t), for different thicknesses of silk, the FDH of parameter $\alpha_{q}$ cannot effectively characterize the orientation and fast axis of the silk fibers.

### 4.2 Robustness Analysis of Three Anisotropic Parameters

The experimental results show that the three parameters used in the work are different in the characterization of fiber long-axis orientation and fast-axis orientation. In this section, we discuss the applicable range of these three parameters combined with theoretical and experimental results.

#### 4.2.1 Difference between θ and $\alpha_{q}$ in detecting fast axis

θ and $\alpha_{q}$ are both parameters to extract the fast-axis information of biological fibers. For pure cylindrical samples [e.g., glass fiber in Fig. 5(a)] or pure birefringent samples [e.g., air-dried tendon in Fig. 5(b)], the fast-axis tracking ability of both parameters is the same. However, for a sample composed of birefringent fibrous scatterers (e.g., silk fiber in Fig. 6), the fast-axis tracking ability of these two parameters is not the same. The bimodal distribution of θ can more effectively reflect the variation of anisotropy within the sample (two perpendicular peaks correspond to the intrinsic birefringence and form birefringence), while the FDH of $\alpha_{q}$ is unstable in tracking the fast axis. The reason for this difference can be found in the mathematical calculation theory of the two parameters.

For the calculation of θ, the measured Mueller matrix M was decomposed into the product of scattering depolarization matrix $M_{\Delta }$, diattenuation matrix $M_{D}$, and phase retardance matrix $M_{R}$ according to MMPD, as shown in Eq. (7). This separates the scattering and diattenuation information in the original Muller matrix M, and the birefringence information is contained in $M_{R}$. Then the parameter θ is calculated from $M_{R}$ according to Eq. (1). For a birefringent cylindrical sample, the vertical bimodal distribution in θ FDH can be seen

$$M=M_{\Delta }M_{R}M_{D}.$$ (7)

For the calculation of $\alpha_{q}$, Eq. (3) directly calculate $\alpha_{q}$ using M42 and M43 elements in the measured Muller matrix M according to MMT, without considering the influence of scattering and diattenuation on these two matrix elements. For a pure birefringent sample (without scattering depolarization and diattenuation), such as QWP, the theoretical Muller matrix of QWP is

$$M_{\mathrm{QWP}}=[\begin{array}{cccc} 1 & 0 & 0 & 0\\ 0 & \cos^{2}\;2\theta & \sin\;2\theta \;\cos\;2\theta & -\sin\;2\theta \\ 0 & \sin\;2\theta \;\cos\;2\theta & \sin^{2}\;2\theta & \cos\;2\theta \\ 0 & \sin\;2\theta & -\cos\;2\theta & 0 \end{array}],$$ (8)

where θ is the direction of the fast axis.

In Eq. (8), $m_{42}=\sin(2\theta )$, $m_{43}=-\cos(2\theta )$. And through Eq. (3), we can clearly see that θ and $\alpha_{q}$ are equivalent. However, when there is multiple polarization information in the sample, $m_{42}$ and $m_{43}$ are the coupling of multiple effects (e.g., scattering and birefringence), and the physical meaning of the two elements is no longer as clear as in Eq. (8). $\alpha_{q}$ will have errors when Mueller matrix contains strong anisotropic linear depolarization,^29^ and it is not reasonable to calculate $\alpha_{q}$ directly through Eq. (3). Therefore, for birefringent cylindrical samples (as shown in Fig. 9), the parameter θ derived from MMPD should be used preferentially to evaluate the fast-axis orientation. Meanwhile, when the Mueller matrix contains weak depolarization and weak diattenuation, $\alpha_{q}$ still can track the fast-axis orientation (as shown in Fig. 5).^29^

#### 4.2.2 Analysis of measurement error of $\alpha_{P}$ and $\alpha_{q}$

**Fig. 10:**
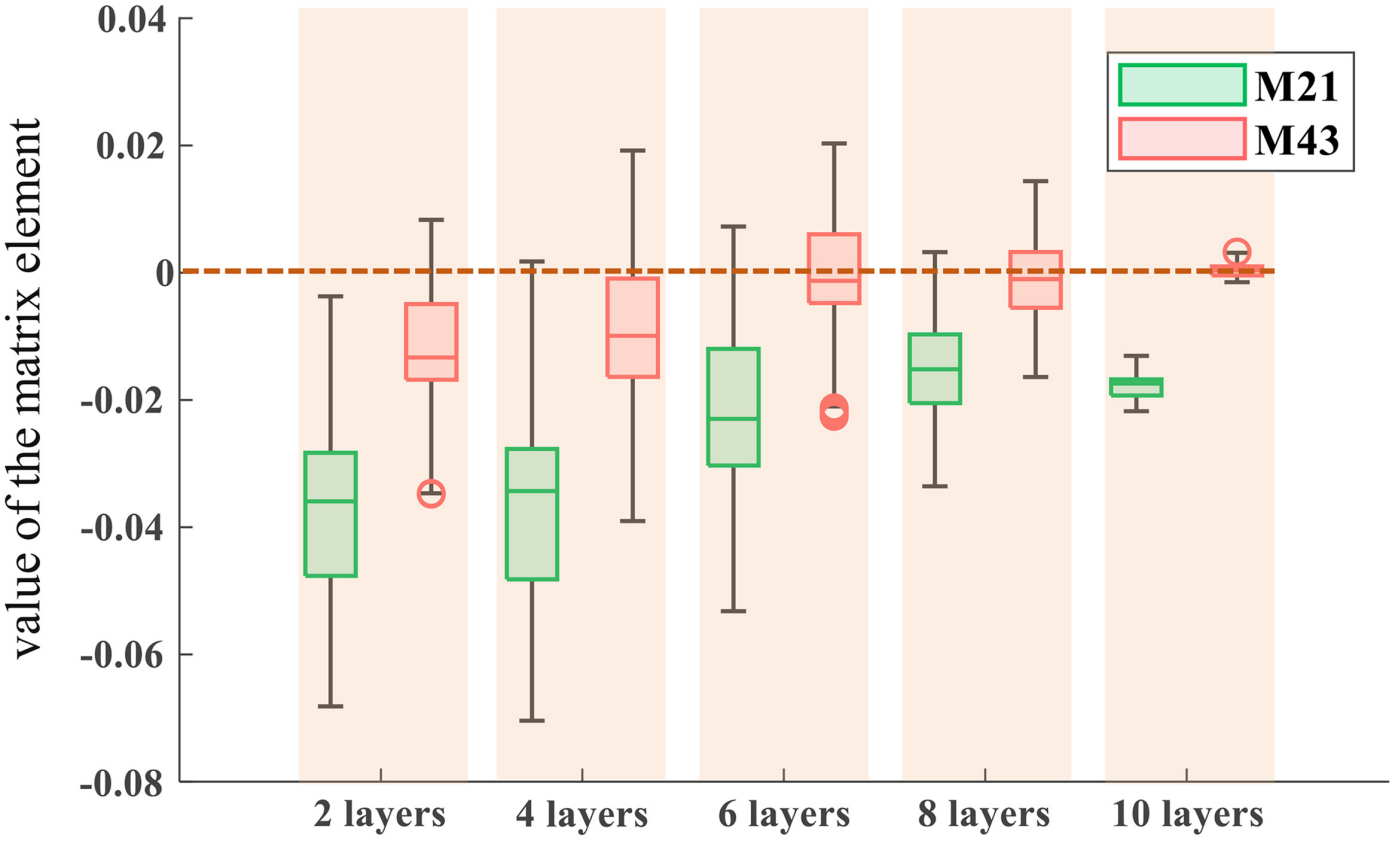
Boxplot of values of $m_{21}$ and $m_{43}$ of neatly arranged silk. The thicknesses of the silk are 2, 4, 6, 8, and 10 layers, respectively.

Based on the experimental results in Sec. 4.1, we analyzed the error sources of $\alpha_{P}$ and $\alpha_{q}$ from the perspective of numerical calculation. In Eqs. (2) and (3), $m_{21}$ and $m_{43}$ are used as denominators respectively, and the closer their absolute values are to zero, the more likely they are to cause large errors. Figure 10 shows the statistical boxplot graph for $m_{21}$ and $m_{43}$ in the Muller matrix corresponding to Figs. 9(a)–9(e). It is obvious that the value of $m_{43}$ is closer to 0, which indicates that $\alpha_{q}$ has a large error. In contrast, the value of $m_{21}$ is far from zero, so $\alpha_{P}$ has relatively high reliability. In biological fibers, $\alpha_{P}$ is mainly associated with cylindrical scattering. As shown in Figs. 5(a) and 6, no matter the cylinder with or without birefringence is, there will be diattenuation caused by cylinder scattering, and $\alpha_{P}$ correspondingly presents a unimodal distribution with its peak angle perpendicular to the actual long axis of the fiber. In Figs. 7(h)–7(l) and Figs. 8(i)–8(l), $\alpha_{P}$ of muscle fibers and tendons also show a robust effect.

### 4.3 Influence of Fibers Stacked in Different Orientations

The superposition of various anisotropies in biological tissues is an important reason for the difficulty in decoupling complex polarization information. Here we discuss three types of anisotropy superimposed on fiber samples: The first type is parallel combination mode (PCM), in which the fiber itself has both form birefringence and intrinsic birefringence. As described in Sec. 3.1.2, silk is a typical birefringent fiber. The second type is serial combination mode (SCM), in which two or more layers of nonbirefringent fibers (such as glass fiber^23^ with different long-axis orientations are stacked. The third type is serial-parallel combination mode (SPCM), in which two or more layers of birefringent fibers (such as silk fiber) with different long-axis orientations are stacked. Here we compare the fast-axis distribution characteristics of the three types.

#### 4.3.1 Fast-axis orientation of PCM

As to PCM, the light scattering and transmission process in the silk sample occur simultaneously, and the FDH of θ shows a vertical bimodal distribution, as shown in Fig. 6. However, previous work tended to average all data of θ to obtain the overall fast-axis orientation of the sample, ignoring the bimodal characteristics of the statistical distribution. If the FDH of θ is a unimodal distribution, the global mean can represent the principal orientation. However, if the bimodal distribution also takes the global average, it may lead to errors in orientation evaluation. Therefore, for birefringent cylinders, it is more accurate to extract information from bimodal statistical distribution characteristics. The experimental results in Secs. 3.2 and 4.1 show that the changes in the two peaks in the bimodal distribution can be used to track the changes in sample microstructure.

#### 4.3.2 Fast-axis orientation of SCM

**Fig. 11:**
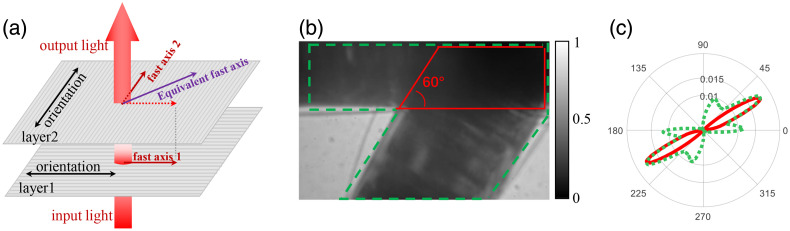
(a) Schematic diagram of light passing through two layers of nonbirefringent cylinders with different orientations. (b) M11 image with two layers of glass fibers stacked at an angle of 60 deg. (c) FDH of θ, where FDH of the green dashed line corresponds to the area surrounded by the green dashed line in (b). FDH of the red solid line corresponds to the area surrounded by the red solid line in (b).

As to SCM, the incident light passes through the glass fiber layers with different orientations in sequence, as shown in Fig. 11(a). In Fig. 11(b), two layers of glass fibers aligned at 60 deg are stacked. The solid red line is the overlapping area of the two layers of glass fibers, and the dotted green line is the overall fiber area, including the overlapping area and two single-layer areas. In Fig. 11(c), the θ FDH in the green dashed line corresponds to the overall fiber region surrounded by the green dashed line in Fig. 11(b), which is reflected as three peaks near 0 deg, 30 deg, and 60 deg. The θ FDH in the solid red line corresponds to the stacked fiber region within the solid red line in Fig. 11(b), which is reflected as a unimodal distribution with a central angle ∼30  deg. These results show the fast-axis orientation differences of SCM and PCM based on polarization analysis. For a layered stacking of nonbirefringent fibers, such as glass fibers, fast axis is suitable for characterizing the general orientation after the integration of multiorientation anisotropy. When the scattering intensity of two layers of fibers is comparable, the final equivalent fast axis is the angular bisector of the fast-axis orientation of the two layers.

#### 4.3.3 Fast-axis orientation of SPCM

**Fig. 12:**
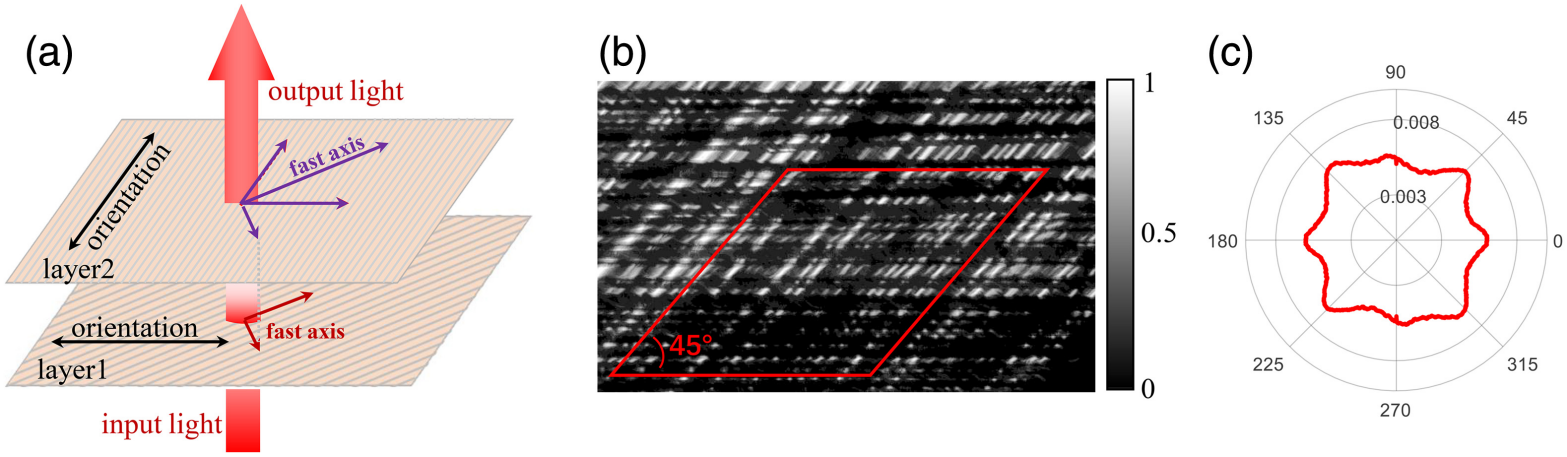
(a) Schematic diagram of light passing through two layers of birefringent cylinders with different orientations; (b) M11 image with two layers of silk fibers stacked at an angle of 45 deg; and (c) FDH of θ, corresponds to the area surrounded by the red solid line in (b).

Few nonbirefringent fibers in biological tissue are as perfect as glass fibers. Given that most fibers are birefringent cylinders (such as muscle and tendon), SPCM is more common in biological tissues. As shown in Fig. 12(a), for birefringent fibrous scatterers like the silk sample, the simultaneous existence of two anisotropic sources in the same layer, mixed with stacked fiber layers with different orientations, can make it difficult to distinguish the specific fast-axis orientation. As shown in Fig. 12(b), we measured two layers of silk stacked at 45 deg. The θ FDH of the sample is shown in Fig. 12(c), and the stacked silk generates an intricate multipetal pattern. This multipetal distribution cannot track the actual fiber orientations, but it can be used as an indicator to assess whether the order of the fiber is broken.

## 5 Conclusion

This work focuses on two possible anisotropic sources in biological tissue samples: intrinsic birefringence by molecular polarity and form birefringence by fiber scattering. By observing and comparing the forward Mueller matrix images of a variety of typical anisotropic phantoms and biological tissues, several orientation related polarization parameters show respective ability to track different anisotropy types. $\alpha_{P}$ shows a strong robust unimodal distribution to track the structural orientation of fibers, whose peak angle is always perpendicular to the fiber long axis. More interestingly, the fibers containing both form birefringence and intrinsic birefringence will show a bimodal distribution on their FDH of θ, which provides a way to evaluate and identify orientation-related tissue properties. The parameters describing the bimodal change of orientation are extracted and their trends with different types of tissue microstructural changes are present. Concretely, this bimodal distribution can only occur on the fibrous structure where anisotropic scattering and material birefringence coexist, totally different from pure birefringent transparent medium, fibers without birefringence and layered and multioriented fibers. Moreover, by analyzing the probability density, the fluctuation range of two peaks, and their relative strength, we can establish a new method to evaluate the changing trend of scattering microstructures in tissues, the stress state of elastic tissues, such as muscle, or explain and identify tissue optical properties based on their difference of anisotropic sources. This preliminary research is expected to show more application values in pathologic diagnosis and quantitative tracking of tissue processes in the future.

## Supplementary Material



## Data Availability

Data underlying the results presented in this paper are not publicly available at this time but may be obtained from the authors upon reasonable request.
