# Supplementary material for: Study on anisotropy orientation due to well-ordered fibrous biological microstructures
Source: J Biomed Opt. 2024 Feb 28;29(5):052919. doi: 10.1117/1.JBO.29.5.052919 (PMC10901243; doi:10.1117/1.JBO.29.5.052919)
Supplement: Supplementary file 1 [file JBO_029_052919_SD001.pdf]

Supplementary Material

Study on anisotropy orientation due to well-ordered fibrous biological microstructures

Zhidi Liu,[a,b] Jiawei Song,[b,c] Qiqi Fu,[a,b] Nan Zeng,[a,b,*] and Hui Ma [a,b,c]

[a]Tsinghua University, Shenzhen International Graduate School, Shenzhen, China

[b]Tsinghua University, Guangdong Research Center of Polarization Imaging and Measurement Engineering Technology, Shenzhen Key Laboratory for Minimal Invasive Medical Technologies, Shenzhen International Graduate School, Shenzhen, China

[c]Tsinghua University, Department of Physics, Beijing, China

Table S1. Values of the depolarization(Δ), diattenuation(D) and linear retardance(LR) of glass fiber.

Long axis orientation	$\Delta \in (0,1)$	$D \in (0,1)$	$LR \in (0^\circ,180^\circ)$
0°	0.19±0.06	0.08±0.04	3.11°±1.34°
45°	0.17±0.05	0.07±0.05	3.37°±1.03°
90°	0.22±0.04	0.09±0.03	3.52°±1.09°
135°	0.21±0.04	0.07±0.03	4.38°±1.12°

[a] Table S1 corresponds to the data in Fig5. (a).

Table S2. Values of the Δ , D and LR of air-dried tendon.

**Long axis orientation**	$\Delta \in (0,1)$	$D \in (0,1)$	$LR \in (0^\circ,180^\circ)$
**0°**	0.19±0.06	0.06±0.03	92.06°±40.86°
**45°**	0.18±0.05	0.07±0.04	99.18°±41.54°
**90°**	0.18±0.05	0.06±0.03	107.87°±44.92°
**135°**	0.17±0.06	0.08±0.05	115.83°±47.51°

[a] Table S2 corresponds to the data in Fig5. (b).

Table S3. Values of the Δ , D and LR of silk fiber.

**Long axis orientation**	$\Delta \in (0,1)$	$D \in (0,1)$	$LR \in (0^\circ,180^\circ)$
**0°**	0.41±0.16	0.08±0.06	6.47°±5.11°
**45°**	0.43±0.16	0.07±0.05	6.26°±4.62°
**90°**	0.29±0.14	0.06±0.05	5.33°±3.01°
**135°**	0.39±0.16	0.04±0.03	4.48°±3.42°

[a] Table S3 corresponds to the data in Fig6.

Table S4. Values of the Δ , D and LR of Chicken breast slice.

Stretched state	$\Delta \in (0,1)$	$D \in (0,1)$	$LR \in (0^\circ,180^\circ)$
CS2	0.51±0.06	0.17±0.03	90.34°±40.08°
CS1	0.55±0.09	0.17±0.02	66.11°±52.96°
NS	0.63±0.04	0.19±0.02	114.81°±53.31°
LS1	0.67±0.04	0.16±0.02	58.23°±46.88°
LS2	0.67±0.05	0.20±0.03	105.08°±41.01°

[a] Table S4 corresponds to the data in Fig7.

Table S5. Values of the Δ , D and LR of skeletal muscle and tendon.

Sample	$\Delta \in (0,1)$	$D \in (0,1)$	$LR \in (0^{\circ},180^{\circ})$
600μm bovine skeletal muscle	0.77±0.04	0.08±0.03	89.01°±54.51°
600μm bovine tendon	0.99±0.01	0.13±0.03	35.21°±14.57°
300μm bovine tendon	0.92±0.02	0.28±0.06	34.07°±32.17°
200μm bovine tendon	0.75±0.05	0.44±0.08	23.68°±20.48°

[a] Table S5 corresponds to the data in Fig8.
